# Leprosy in Minnesota

**Published:** 1884-02

**Authors:** Chas. Grönvold

**Affiliations:** Norway, Minn., Member of State Board of Health of Minnesota, and Chairman of its Standing Committee on Leprosy; Norway P. O., Goodhue Co., Minn.


### ARTICLE V.

LEPROSY IN MINNESOTA. Report to the State Board of Health, of Minnesota, by DR. CHR. GRÖNVOLD, of Norway, Minn., Member of State Board of Health of Minnesota, and Chairman of its Standing Committee on Leprosy.*

There has lately been written much about a center of leprosy in the Northwest, from which the disease is said to be spreading. It may be interesting to see, how facts support this statement.

As early as in 1864, the condition of the lepers among the immigrants to the northwest had been an object for the attention of medical men from their old country.

In that year Dr. J. H. Holmboe, surgeon in charge of the Hospital for Lepers in Bergen, Norway, visited this country to study the influence that change of climate and other relations had on the development of the disease. He found twelve cases, of which two had originated in this country, while one, which came here leprous, had got well. His impression was, that their condition of health was better, than it would have been if they had staid in the old country.

In 1869-70 the settlements were again visited by a medical gentleman from the old country, the late Professor William Boeck, of Christiania, Norway, well known in medical literature for his writing on leprosy and syphilis. The result of his investigations in the Northwest Settlements may be read in *Nordiskt Medicinskt Archiv*, Band III., No. 1. He found in the three States, Wisconsin, Iowa and Minnesota, eighteen cases of leprosy, all of which had come from those parts of the western Norwegian seacoast, where the disease is endemic. Nine cases had the anæsthetic form, three the tubercular, while in six

---

*This article was furnished to this Journal as an "Original Communication," but for want of space was for more than one month deferred publication. It has since appeared in the *Journal of Cutaneous and Venereal Diseases*, but we are pleased to present it in the deparment for which it was originally intended as a contribution which can not fail to interest our readers in the North-West.

cases both forms were represented at the same time, or one superseding the other. In regard to the time of the appearance of the disease, in nine it had commenced already in the old country, and of these, five had lepers in their family, while four did not know of any leprous relations.

In the other nine cases the disease first commenced in this country. Of these, eight had lepers in their family, and the disease broke out respectively, two and a half, three, three and a half, five, six, eight, nine and a half and ten years after their arrival. These eight cases, that were developed on American soil, may depend on heredity, or they may have been caused by contagion in the old country, the only place, where it is probable that they could have met with other lepers, or the disease may have developed from a miasma in the old country. Either of the last two suppositions being right, the time of incubation will for two of the cases be nine and a half and ten years. Prof. Boeck considers them all as depending upon heredity in direct or lateral line. If he had any doubt, he says, in regard to the propagation of the disease by heredity, these cases would have convinced him.

As an instance, here is one of the cases, described in the above named *Medicinskt Archiv*.

"S. S., 45 years old, came, fourteen years ago, to America, to the place, where he now resides, and was in every respect perfectly well. His father had died, about 50 years old, in the Lepers' Hospital at Bergen; his brother and sister died leprous in the old country, one and four years ago; his father's sister had also had the disease. S. continued to be in good health, after his arrival to this country, for nine and one-half years. Four and one-half years ago he commenced feeling heavy and drowsy, and two years later, after a violent cold, tearing and fleeting pains announced themselves in the hands and feet, and also in the upper and lower extremities, and at the same time there was swelling of the same parts and the face, and an eruption of red spots appeared. The pains were so violent that he was obliged to stay in the house from January to May, and when he was able to get out of bed, his strength was gone, and he discovered, that his sensation was very much lessened along the peroneal side of the feet and legs, and on the ulnar side of the hands and arms. Since that time he has always suffered from fleeting pains in the extremities and feels heavy and drowsy. 'I will never have a day of health any more,' he said. Sensation is at present very weak in hands and feet, as well on the outer as on the inner side, from the hands along the arms to the shoulders, and from the feet along the legs and thighs to the neighborhood of the hip. The muscles of the right hand, between the first and second finger, and those of the fifth finger, are considerably wasted."

This patient left the old country in good health, and continued to be so for nine and one-half years after his arrival in this country, in a place where there were no lepers before, and where it is not known that he has had communication with any.

The last of the nine cases, developed on American soil, did not know of any leprous relation, and may possibly be referred to contagion. The case is, in short, as described in the above named *Archiv*:

"B. J. is 39 years old; came to this country fourteen years ago; has for twelve years suffered, and is yet suffering from rheumatic pains in the extremities. Recently, after a day's hard work, she had in the evening a violent chill, and the next day there appeared spots on the arms, and successively on the lower extremities, chest, back, and face. These spots are distinctly morphœa spots, in some places slightly elevated. In them there is either complete anæsthesia, or very little sensation; outside of them the sensation is normal, except on the external side of the feet and legs. Her appearance is very good. She is fleshy, has good appetite; the bowels are a little constipated; menstrual flow scanty. She does not know of any leper in the family. She has once, six or seven years ago, been in the home of a leper. By others, it is said, that she, for some time, has taken care of a leper."

Prof. Boeck had his doubts whether this person did not also belong to a leprous family, and whether the disease was not due to heredity.

The professor was of the opinion that lepers here are better off as regards their disease than they would have been in the old country. "They have come away from the place where we see leprosy may originate spontaneously, and which certainly will favor its development, when the disposition thereto is inherited. They have settled on fertile lands, where they certainly have to work hard to make a living, but they generally undergo no hardships, as we in Norway understand the term. Here is no work that can be compared with that done at the midwinter fisheries in open sea off the Finmark coast, or the hardships suffered while tending the cattle on the high mountain plateaus, which causes, so often, bring out the latent leprosy."

Since Prof. Boeck's visit in 1869–70, investigations have been continued in Minnesota regarding the occurrence and character of the disease in that state.

At present six cases and their whereabouts are known, all emigrated from that part of western Norway, where the disease is endemic. None of them are confined to bed, and most of them are able to perform their daily duties.

The following table will show some of the particulars of the cases:

| | | Age. | How long leprous. | How long in this country | Form of disease. | Leprous Relations. |
|---|---|---|---|---|---|---|
| Case I.* | Male | 58 years old | 18 years | 27 years | Anæsthetic | Father, and father's sister, brother, sister, cousins |
| Case II. | Male | 29 " " | 7 " | 20 " | Tubercular | Father, two brothers |
| Case III. | Female | 29 " " | 7 " | 16 " | Tubercular | None |
| Case IV. | Male | 35 " " | 16 " | 12 " | Anæsthetic | None |
| Case V. | Male | 67 " " | Prodromes in the Old Country | 17 " | Anæsthetic | None |
| Case VI. | Male | 44 " " | 10 years | 15 " | Anæsthetic | Probably a brother |

*Case No. I. is described by Prof. Boeck as No. 1., and is the one described above as "S.S." It is the only case, yet alive, of them he saw in Minnesota. He has, since the Professor saw him, been tolerably well, until the latest years, when the disease has made a quicker advance.

It will be seen, that two of them have the tubercular form, while four have the anæsthetic; in two of these—IV. and VI.—a little complicated with the tubercular. Two of them—IV. and V.—had the disease in the Old Country; but of these, one did not suffer much from it before seven years after the arrival to this country.

Of the other four, three know of leprous relations, while one—III.—denies that the disease has ever been in her family. She is from a district where there are many lepers--Balestrand in Sogn. The main features of the case are:

Case III. came to America when she was 13 years old, in company with a sister of her mother, sixteen years ago, and has all the time since lived near the place she first went to. The first two years she lived with her aunt, but, after confirmation, she served as housemaid in families of the neighborhood, until she, seven years ago, discovered the first signs of the disease. After a severe illness, she says caused by cold, the legs swelled and some vesicles appeared, first on one, later on both ankles, followed by sores.

At present she exhibits the tubercular form of the disease, and presents a very repulsive appearance. The complexion is pale, or rather dirty white, the skin of the face thickened and rough. Tubercles, some suppurating, some torn by scratching, are seen over the eyes and on other places of the forehead, on the right nostril, and on the cheeks and chin; also on the ulnar side and back of both hands, where, as well as on the outer side and back of the feet and lower part of the peroneal side of the legs, is some anæsthesia. The voice is hoarse and rough. The nose seems flattened on account of the alæ spreading out. The lobes of the ears and the lips, especially the upper, are very much thickened and elongated. The eyebrows and eyelashes are gone, conjunctiva dirty white, with a troublesome secretion; cornea somewhat opaque; her eyesight is very much impaired. She denies having any discoloration of the skin, any sores or tubercles in other places than those above named, and on the ankles, where there are deep ulcers, laying the bone bare. She does not suffer much; only once in a while she feels a little oppression, and some slight pain in the chest and back; but this, she believes, is only caused by weakness.

Appetite is irregular, the sleep is commonly good. Menses commenced to be scanty when the disease broke out, and stopped soon. She feels worse in the spring and fall, but is seldom confined to bed. She takes cold easily. Like almost all lepers here, she ascribes her disease to cold. She suffered so from cold, she explains, in a place, where she was working, that she then and there got the disease. It is not known by herself or anybody else, that she ever met with a leper in this country. She must have carried the germ of the disease with her from the old country, whether it has been communicated by contagion or a miasma, if she is right in her statement that the disease has been neither in her father's nor mother's family. The disease became evident when she was twenty-two years old, and had lived in this country nine years. She will not remember any prodromes; has probably tried to conceal her condition as long as possible. She tries yet to put the best appearance on her miserable condition.

Ten lepers of the immigrants are known to have died in Minnesota since the settlement of the conntry, all of them males. Of these seven died in the last seven years, and the following table will show some of the particulars of the cases :

| | Age at time of death. | How long leprous | How long in America. | Form of the disease. | Leprous Relations. |
|---|---|---|---|---|---|
| Case I. | 49 years old | 24 years | 19 years | Anæsthetic | Brother of mother's father |
| Case II.* | 56 " " | 14 " | 24 " | Tubercular | A cousin |
| Case III. | 35 " " | 10 " | 9 " | Anæsthetic | Father's brother |
| Case IV. | About 30 years old | 3 " | 13 " | Tubercular | Brothers. Father and brother diseased |
| Case V. | About 30 years old | 12 " | 15 " | Tubercular | |
| Case VI.* | 62 years old | 30 " | 21 " | Tubercl. first 7 years, afterwards anæsthetic; disease stopped before death | Brother and father's brothers |
| Case VII. | 30 " " | | 10 " | Anæsthetic | Mother's brother |

*Cases II. and VI. were seen by Prof. Boeck in 1870, and described by him in the above named journal, as observation 3 and 2. Case II. got steadily worse until death, eight years after the Professor saw him, but in No. VI. the disease made no advance after the time Prof: Boeck saw him. He was in good health for many years, with the exception of the anæsthesia in the places already attacked, and an always open and discharging ulcer under one foot. When this discharge stopped, about fourteen days before his death, vomiting set in, which became more and more distressing, so he could keep nothing in his stomach. He was not confined to bed, but moved about as usual, until the day and hour of his death, which occurred thirty years after he was attacked, seven years after Prof. B. saw him and described his case.

It will be seen that three of them had the anæsthetic form, and died twenty-four, ten and thirty years after they got the disease. The last case, No. VI., Boeck's observ. 2, is an instance of one form changing into the other; the disease commenced in the tubercular form, and continued so for seven years; after a violent fever, it changed into the anæsthetic. Three had the tubercular form, and died, after having had the disease fourteen, three, and twelve years. Case II. got the disease ten years after his arrival in this country, when he was 42 years old.

All of them had lepers in their family ; three of them had the disease in the old country ; three got it here ; two, ten years, and one, three years after their arrival. Four of the lepers—two of them living yet—have full grown children, born in America ; two have also full grown grandchildren. One (No. VI. of last table—Boeck's observ. No. 2) has fifteen grandchildren, aged from 2 to 22, and two great-grandchildren, and in none of them has any sign of the disease been discovered. The other (No. 1 of the first table, Boeck's observ. No. 1), who belongs to a family so strongly infected with leprosy—lepers were his father and father's sister, his brother and his sister—has full grown children, and many grandchildren of an age up to 17, all in good health.

The suggestion of the above facts, as far as they go, seems to be, that the disease is not so easily acquired here in the Northwest as in the old country, be it by heredity or contagion. The dry climate is possibly not so favorable for the development and the communication of the disease, that at present mostly belongs to the sea coasts and islands. The chances of contagion are decidedly less here than in the old country ; there is greater cleanliness, as a consequence of greater economical prosperity, and the new built houses of the first settlers in a new country furnish no filthy nests for contagion.

But once acquired, the disease seems to run its regular course without abatement.

Norway P. O., Goodhue Co., Minn.

